# Genetic Diversity and Population Structure of Cowpea [*Vigna unguiculata* (L.) Walp.] Germplasm Collected from Togo Based on DArT Markers

**DOI:** 10.3390/genes12091451

**Published:** 2021-09-20

**Authors:** Kodjo M. Gbedevi, Ousmane Boukar, Haruki Ishikawa, Ayodeji Abe, Patrick O. Ongom, Nnanna Unachukwu, Ismail Rabbi, Christian Fatokun

**Affiliations:** 1 Cowpea Breeding Unit, International Institute of Tropical Agriculture (IITA), PMB 5320, Oyo Road, Ibadan 200001, Oyo State, Nigeria; o.boukar@cgiar.org (O.B.); h.ishikawa@cgiar.org (H.I.); p.ongom@cgiar.org (P.O.O.); n.unachukwu@cgiar.org (N.U.); i.rabbi@cgiar.org (I.R.); c.fatokun@cgiar.org (C.F.); 2 Life and Earth Sciences Institute (Including Health and Agriculture), Pan African University, University of Ibadan, Ibadan 200284, Oyo State, Nigeria; 3 Department of Crop and Horticultural Sciences, University of Ibadan, Ibadan 200284, Oyo State, Nigeria; ayodabe@yahoo.com

**Keywords:** cowpea, germplasm, genetic diversity, population structure, DArT markers

## Abstract

Crop genetic diversity is a sine qua non for continuous progress in the development of improved varieties, hence the need for germplasm collection, conservation and characterization. Over the years, cowpea has contributed immensely to the nutrition and economic life of the people in Togo. However, the bulk of varieties grown by farmers are landraces due to the absence of any serious genetic improvement activity on cowpea in the country. In this study, the genetic diversity and population structure of 255 cowpea accessions collected from five administrative regions and the agricultural research institute of Togo were assessed using 4600 informative diversity array technology (DArT) markers. Among the regions, the polymorphic information content (PIC) ranged from 0.19 to 0.27 with a mean value of 0.25. The expected heterozygosity (He) varied from 0.22 to 0.34 with a mean value of 0.31, while the observed heterozygosity (Ho) varied from 0.03 to 0.07 with an average of 0.05. The average inbreeding coefficient (F_IS_) varied from 0.78 to 0.89 with a mean value of 0.83, suggesting that most of the accessions are inbred. Cluster analysis and population structure identified four groups with each comprising accessions from the six different sources. Weak to moderate differentiation was observed among the populations with a genetic differentiation index varying from 0.014 to 0.117. Variation was highest (78%) among accessions within populations and lowest between populations (7%). These results revealed a moderate level of diversity among the Togo cowpea germplasm. The findings of this study constitute a foundation for genetic improvement of cowpea in Togo.

## 1. Introduction

Cowpea [*Vigna unguiculata*, (L.) Walp.], an annual herbaceous legume plant, is widely distributed in tropical and subtropical regions of sub-Saharan Africa (SSA), where it plays important roles in both human nutrition and food security, income generation for farmers and food vendors, and feed for livestock. Cowpea grains are rich in protein (23.0% to 32.0% depending on variety), carbohydrates and folic acid, and contain considerable amounts of some minerals [1]. The young leaves are used as spinach in eastern and southern Africa, while the green immature pods and green mature seeds are consumed in Senegal and some other African countries [2].

Cultivated cowpea, which belongs to the subspecies *unguiculata*, is divided into five cultivar groups, namely Unguiculata, Sesquipedalis, Textilis, Biflora, and Melanophthalmus [3,4]. The commonly cultivated cowpea belongs to cultivar group Unguiculata. Cowpea is diploid with 2n = 22 and a genome size of about 620 million base pairs [5,6]. The crop is autogamous, but up to 5% outcrossing has been reported in the cultivated varieties, probably due to insect activities [7].

From 2014 to 2018, the world annual average cowpea production was 6.57 million tons on a harvested area of 12.4 million ha with an average yield of 0.53 t/ha. Africa, and specifically West Africa, produced 96% of the world production on 82.8% of the land area [8]. Worldwide, Nigeria is the largest producer of cowpea followed by Niger, Burkina Faso, Cameroon, and Mali [6]. In Togo, cowpea is grown from the moist to dry zones singly or in a variety of crop mixtures. Between 1980 and 2013, cowpea production in Togo increased from 19,630 tons to 132,636 tons with the highest production in 2012 [9]. Cowpea yield in Africa is low compared to Asia and the United States of America [6,10]. This low productivity is attributable to a wide range of factors, which include a number of abiotic (drought, heat, low soil fertility) and biotic (insects, diseases, parasitic weeds, low yields of farmers’ varieties) factors [6]. Landraces are a repository of crop diversity that have evolved through natural and artificial selection over millennia and represent valuable resources for crop adaptation to stresses [11].

Genetic diversity of crops plays an important role in sustainable development and food security, as it serves as a source of genes needed in the development of better performing and well adapted varieties [12]. Food production and security depend on the conservation and wise use of agricultural biodiversity.

Several approaches have been utilized to enhance our knowledge of the nature and extent of variability among cowpea accessions stored in different genetic resource centers. To evaluate the genetic diversity of a given crop, morphological (phenotype) and molecular (genotype) markers have been used. However, morphological attributes are subject to environmental influences, may vary at different developmental stages, and are limited in number [13]. Molecular markers are superior to morphological parameters by being present in abundance in organisms and neutral to environmental effects. Several studies have been carried out on genetic diversity of cowpea using different types of genetic markers. Ba et al. [14] used RAPD markers to characterize the genetic variation in domesticated cowpea and its wild progenitor. In that study, twenty-six domesticated accessions representing the five cultivar groups of *Vigna unguiculata* ssp. *unguiculata* and 30 wild/weedy accessions, which included those from West, East and Southern Africa, were evaluated. The wild accessions from East Africa were most diverse [15]. Ajibade et al. [16] used inter simple sequence repeat (ISSR) DNA polymorphic markers to study the genetic relationships among 18 *Vigna* species. Diouf and Hilu [17] assessed the genetic diversity among cowpea accessions and varieties from Senegal using microsatellite and RAPD markers and reported the superiority of microsatellite markers in detecting relationships. Badiane et al. [18] further analyzed the genetic variation among Senegalese cowpea germplasm using microsatellites and reported clustering in the same group of most of the local varieties, suggesting their close relationship compared to the breeding lines. Using single nucleotide polymorphism (SNP) markers, Egbadzor et al. [19] characterized 113 cowpea accessions comprising 108 from Ghana and five from elsewhere. The study showed that SNP markers were more efficient in discriminating among the cowpea germplasm than the morphological, seed protein polymorphism and simple sequence repeat (SSR) markers reported earlier on the same lines. Xiong et al. [20] assessed the genetic diversity and population structure of 768 cultivated cowpea genotypes from USDA GRIN cowpea germplasm, originally collected from 56 countries using genotyping by sequencing. The authors reported three well differentiated genetic populations, a result similar to that of Fatokun et al. [21] on a mini-core of 298 landraces using SNP markers. Recently, a new type of marker platform, diversity arrays technology (DArT), was developed as a novel method for whole genome profiling without the need for sequence information. It is a high-throughput method able to discover hundreds of markers in a single experiment, at low cost per data point [22]. Potential applications of DArT include germplasm characterization, genetic mapping and gene tagging, molecular marker-assisted breeding and tracking of genome methylation changes [23]. The DArT has been successfully deployed in many crops such as wheat [24,25], garlic [26], apple [27], barley [28] and sorghum [29]. In cowpea, DArT markers have been used to map quantitative trait loci (QTLs) for yield associated traits among F_2:3_ populations, and new lines that might combine the attributes of the two parents could be identified [30]. Sodedji et al. [31] assessed the genetic diversity and population structure of 274 cowpea accessions collected from 33 different countries using DArT. The authors reported the presence of three clusters among the germplasm lines. Additionally, Ketema et al. [32] reported three well differentiated genetic populations among 357 cowpea accessions from Ethiopia using DArT markers. The study further showed that accessions from the same region of the country were distributed into different clusters. To date, only a study investigating the genetic diversity among 70 cowpea accessions from Togo using SSR markers has been reported [33]. In this study, DArT markers were applied to 498 cowpea accessions from Togo in order to study genetic diversity and population structure.

## 2. Materials and Methods

### 2.1. Plant Materials

The cowpea accessions were collected from the Republic of Togo between October and November 2018 through the country’s agricultural extension service ICAT (Institut de Conseil et d’Appui Technique) and the agricultural research institute ITRA (Institut Togolais de Recherche Agronomique). The cowpea lines were obtained directly from farmers across 63 locations in the country’s five administrative regions (Figure S1): “Region des Savanes (RS)”, “Region de la Kara (RK)”, “Region Centrale (RC)”, “Region des Plateaux (RP)” and “Region Maritime (RM)” with support from ICAT, while some were received from the cowpea management unit of ITRA. Each of the accessions collected through ICAT was coded using an abbreviation of the name of the region of collection (e.g., RS for Region des Savanes) followed by a serial number, while the names officially assigned to accessions collected from ITRA were used as received. The accessions were transferred to the International Institute of Tropical Agriculture (IITA), Ibadan, Nigeria in December 2018 and kept in cold storage until planting. A total of 520 accessions were planted for seed multiplication at IITA Ibadan in January 2019, but only 498 accessions, comprising 399 from the five regions (105 from “Region des Savanes”, 98 from “Region de la Kara”, 50 from “Region Centrale”, 108 from “Region des Plateaux”, 38 from “Region Maritime”) and 99 from ITRA, were used for this study as the others failed to flower and set pods.

Seeds were harvested from a single plant of each accession. Five (5) seeds of each representative accession were sown in pots containing about 5 kg of topsoil on 25 January 2020 in the screenhouse at IITA and thinned to two plants two weeks after planting and later to one plant per pot. A newly expanded young trifoliate leaf was collected from each plant five weeks after sowing and placed in small plastic bag containing two small packs of previously oven dried silica gel. All samples were kept inside an air-conditioned room for two weeks to allow the leaves to dry.

The dried leaves were later squeezed and a quantity of 10 to 15 mg for each accession was placed in a sampling tube arranged inside six 96 well collection plates. These were shipped to Diversity Arrays Technology Pty. Ltd., (Canberra, Australia).

### 2.2. DNA Extraction and Genotyping

Total genomic DNA extraction was performed at the Diversity Arrays Technology (DArT) facility, Australia, following their in-house DNA extraction protocol (https://www.diversityarrays.com/orderinstructions/plant-dna-extraction-protocol-for-dart/ (accessed on 26 March 2021)). The DNA quality of each sample was qualitatively and quantitatively assessed on 0.8% agarose gel and Nanodrop 2000c spectrophotometer (Thermo Scientific, Waltham, MA, USA), respectively.

Genotyping was done using DArTseq methodology (https://www.diversityarrays.com/technology-and-resources/dartseq/ (accessed on 26 March 2021)). Genomic DNA Library construction was done using genomic complexity reduction technology [34]. Library purification and quantification for cluster generation was done using an automated clonal amplification system (cBOT Illumina) followed by Next Generation Sequencing (NGS) on Illumina Hiseq2500/Novaseq with 1,200,000 reads per sample. The reads were aligned to the cowpea IT97K-499-35 reference genome [35] *Vigna unguiculata* v1.1, publicly accessible on Phytozome (https://phytozome.jgi.doe.gov/pz/portal.html#!info?alias=Org_Vunguiculata_er (accessed on 26 March 2021)).

### 2.3. SNP Filtering

Genotyping outputs were received from DArTseq in HapMap format with a total of 10,671 SNPs. From the first filtering based on 20% missing data, 7998 SNPs were obtained. Following a second filtering by imputation to remove SNPs with major allele frequency (MAF), more than 95% and less than 5% minor allele frequency (MnAF) in TASSEL 5 [36], a total of 4600 informative SNP markers were obtained and used in this study.

### 2.4. Identification of Dupplicates

Pairwise identity by state (IBS) was calculated among accessions, and those with values > 0.98 were considered duplicates. A single accession was retained for each group of duplicated accessions, leading to a dataset of 255 accessions used for further analyses.

### 2.5. Genetic Diversity Parameters

The minor allele frequency (MnAF), major allele frequency (MAF), gene diversity (GD) or expected heterozygosity (He), observed heterozygosity (Ho), polymorphic information content (PIC) and inbreeding coefficient (F_IS_) for each locus were calculated for DArT markers using PowerMarker 3.25 [37].

### 2.6. Analysis of Molecular Variance and Genetic Differentiation

To estimate the explained genetic variation values between areas of collection, analysis of molecular variance (AMOVA) and genetic differentiation (F_ST_) were performed using GenAlEx 6.5 [38]. For the analysis, SNP data were numerically coded as follows: A = 1, C = 2, G = 3, T = 4, while missing data was coded as 0.

### 2.7. Cluster and Principal Coordinate Analyses

A Ward’s minimum variance hierarchical cluster dendrogram was built on the 255 accessions using the Analyses of Phylogenetics and Evolution (ape) package [39] implemented in R [40]. Principal coordinate analysis (PCoA) was performed using GenAlEx 6.5 [38] and plotted using R [40]. Analysis of molecular variance and genetic differentiation were performed on the identified clusters.

### 2.8. Population Structure

The population structure of the 255 accessions was inferred using the Bayesian clustering method implemented in the software STRUCTURE version 2.3.4 [41]. The software was run with the admixture model and correlated allele frequencies. Five runs were performed for each value of K (1 to 10) representing the number of clusters considered. The burn-in number and iterations for each run were both set to 10,000 and the best K value with the highest likelihood for estimating a suitable population size for the dataset was determined using the method described by Evanno et al. [42] using a structure harvester [43]. The most appropriate K value that is useful and better describes the data and also has good correspondence with the grouping pattern obtained by cluster analysis was selected. Cowpea accessions with membership probabilities (inferred ancestry) ≥ 0.80 were assigned to the corresponding groups. All accessions with membership probabilities < 0.80 were assigned to the mixed group. Nei’s genetic distance values obtained from structure output were used to measure divergence between the gene pools.

## 3. Results

### 3.1. Characterization of the SNP Markers

The 4600 DArT markers used in this study were subjected to genetic analysis and six SNP types (Table 1) were determined from them as follows: 1336 (29.04%) A/G, 1294 (28.13%) C/T, 547 (11.89%) A/T, 501 (10.89%) G/T, 465 (10.11%) A/C and 457 (9.94%) C/G SNP types. A higher transition-type SNP level (57.17%, 2630) was observed compared to transversion-type SNPs (42.83%, 1970) in the cowpea germplasm genomes, thus giving a ratio of 1.34:1.

### 3.2. Identification of Duplicates

**Figure 1:**
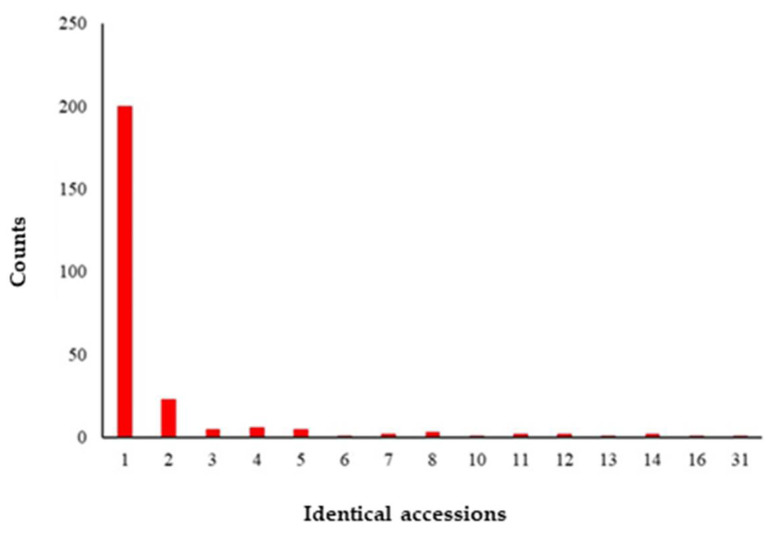
Identical accession groups (duplicates) statistics.

The number of accessions with 98% IBS or higher similarity with another ranged from one (the most common) to 31 individuals (Figure 1). Each group of duplicates was represented by one member while the others were not considered for analysis performed on the unique 255.

### 3.3. Genetic Diversity

Estimated genetic diversity indices show that MnAF ranged from 0.15 for RS to 0.26 for RM with a mean of 0.22 among the five areas of collection and ITRA. The mean MAF was 0.78 across all the accessions, while the mean values for the five regions and ITRA ranged from 0.74 (RM) to 0.85 (RS). The He ranged from 0.04 to 0.50 (mean = 0.31) while the Ho ranged from 0.00 to 0.91 (mean = 0.05) across the 255 accessions. Mean He observed among regions and ITRA ranged from 0.22 (RS) to 0.34 (RM and ITRA), while the Ho ranged from 0.03 (RK) to 0.07 for ITRA. The highest Ho (0.07) among places of collection was observed among the accessions received from ITRA. The PIC ranged from 0.04 to 0.38 (mean = 0.25). Among the regions and ITRA, the PIC varied from 0.19 (RS) to 0.27 (RP, RM, ITRA). The inbreeding coefficient (F_IS_) ranged from −0.83 to 1.0 (mean = 0.83). Among the regions and ITRA, the variation of F_IS_ ranged from 0.78 (ITRA) to 0.89 (RK) (Table 2).

### 3.4. Analysis of Molecular Variance and Genetic Differentiation

An AMOVA was performed on the 255 accessions for the distribution of genetic variation between and within the groups. The results presented in Table 3 revealed significant (*p* < 0.001) variation between populations, accessions and within accessions. Variation among individuals accounted for 78% of the total variation. This was followed by variation within individuals and between populations at 15% and 7%, respectively. The average accession differentiation was 0.072, while the mean value for the inbreeding coefficient and gene flow were 0.836 and 3.21, respectively.

The F_ST_ values were determined to quantify population differentiation among the accessions based on where they were collected from. In the present study, F_ST_ ranged from 0.014 (between RK and RC) to 0.117 (between RS and RM). Generally, weak differentiations were observed among populations. However, some moderate differentiations (0.05 < F_ST_ < 0.15) were observed between ITRA and RK (0.055), RS and ITRA (0.067), RS and RM (0.117), RS and RP (0.055), RM and RC (0.067) and RK and RM (0.091) (Table 4).

### 3.5. Phylogenetic Relationship and Principal Coordinate Analyses

**Figure 2:**
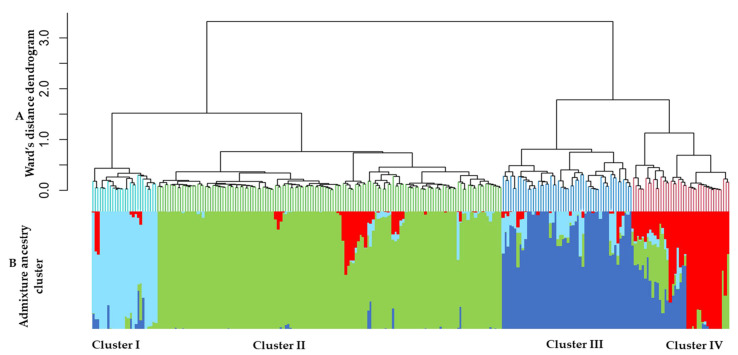
(**A**) Phylogenetic tree of 255 cowpea accessions based on Ward’s method. (**B**) Population structure classification of 255 cowpea accessions using membership probabilities.

A phylogenetic tree resulting from cluster analysis based on Ward’s method performed on the 255 accessions shows four clusters with 26, 138, 52 and 39 accessions in cluster I, cluster II, cluster III and cluster IV, respectively (Figure 2A). Each of the four clusters is composed of a varying number of accessions from all five different locations and ITRA. Cluster I contains 10.2% of the accessions with 3, 7, 7, 1, 4, and 4 accessions, respectively from RS, RK, RP, RM, RC and ITRA. Cluster II contains 54.1% of the total number of accessions, of which 56, 33, 24, 4, 11 and 10 were obtained from RS, RK, RP, RM, RC and ITRA, respectively. Accessions from RS and RK make up 64.5% of this cluster. Cluster III with 52 accessions has 20.4% of the total number of accessions. Twenty of the accessions were from RP and eight from ITRA, implying that about 53.8% of the accessions in cluster III were obtained from these two sources. Of the 39 accessions in cluster IV, 10, 2, 9, 2, 2 and 14 were collected from RS, RK, RP, RM, RC and ITRA, respectively. Accessions from RS and ITRA represented 61.5% of the cluster.

**Figure 3:**
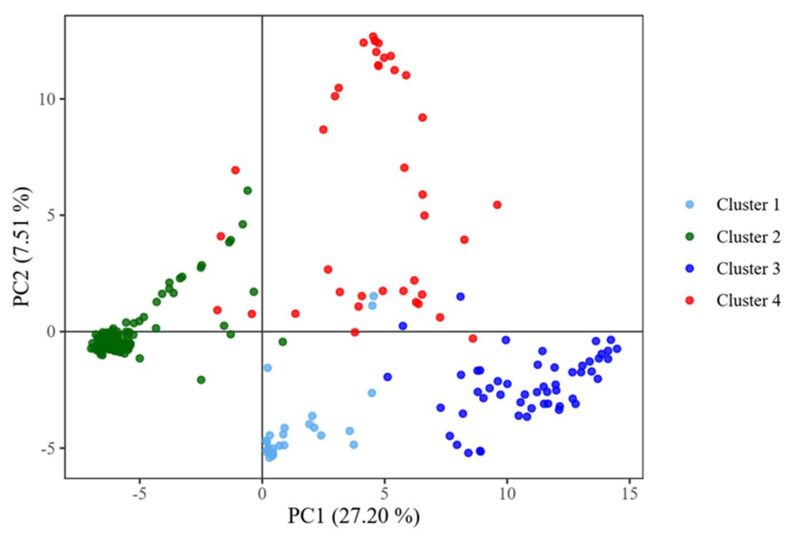
Scatter of the accessions along the first two principal coordinates PC1 and PC2.

The scatter of the cowpea lines along the first two principal coordinates showed that accessions belonging to the same cluster tend to be closely located along the axes (Figure 3). The first and second principal coordinates are responsible for 27.20% and 7.51% of the variation, respectively. The members of the cluster III are most compact in distribution, while those of cluster IV are the most widely distributed along the axes of the first two principal coordinates.

An AMOVA was performed on the accessions for the distribution of genetic variation between and within the four clusters. The results presented in Table 5 reveal significant (*p* < 0.001) variations between clusters, accessions and within accessions. Variation among accessions accounted for 45% of the total variation. This was followed by variation between clusters and within accessions at 42% and 13%, respectively. The average genetic differentiation for the four clusters was 0.423, while the mean value for inbreeding coefficient (F_IS_) and overall fixation index (F_IT_) were 0.774 and 0.870, respectively. Gene flow between the four clusters was 0.381.

GenAlEx 6.5 was used to calculate the genetic differentiation among the four clusters identified in the phylogenetic tree. The pairwise F_ST_ values ranged from 0.140 between clusters III and IV to 0.290 between clusters II and III (Table 6). Apart from the moderate genetic differentiation observed between clusters III and IV, the remaining pairwise F_ST_ values among clusters show high genetic differentiation.

### 3.6. Population Structure

**Figure 4:**
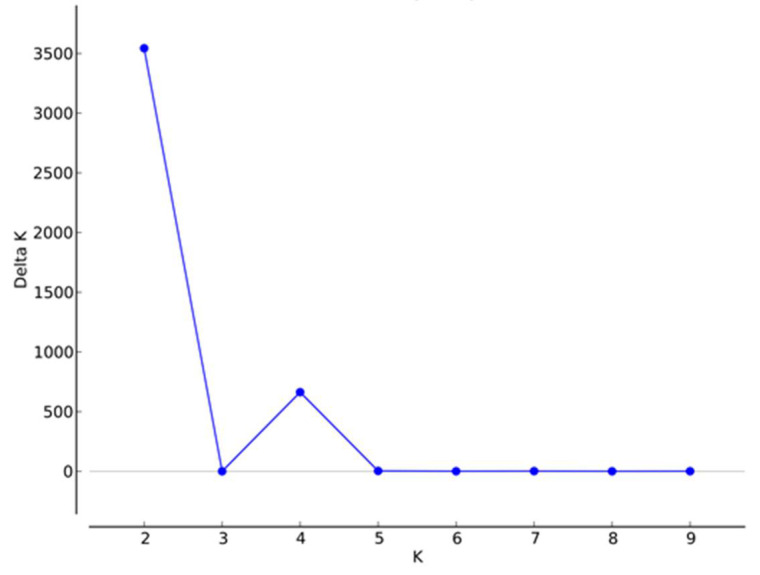
Delta K (ΔK) plot calculated from K = 2 to K = 9.

Population structure analysis was inferred on the 255 accessions. The highest value of delta K (ΔK) was obtained for K = 2, revealing the existence of two gene pools of cultivated cowpea from Togo (Figure 4). Another peak was observed at K = 4, indicating the presence of further subgroups, regarded as gene pools G1, G2, G3 and G4 within the collected cowpea samples. The grouping at K = 4 showed good concordance to that of the dendrogram (Figure 2B). Using a membership coefficient of 0.80, the total of admixed accessions in the K = 4 model is 67, corresponding to 26.3%. In this model, the first gene pool (G1) contains 21 accessions that belong to cluster I. The second gene (G2) pool is composed of 121 accessions from cluster II. The third gene pool (G3) contains 32 accessions that belong to cluster III. The fourth gene pool (G4) is composed of 14 accessions belonging to cluster IV (Table 7).

The genetic divergence among the four gene pools shown by Nei’s net nucleotide distance indicated that G2 and G3 (0.320) are the most distantly related followed by G1 and G4 (0.289). The lowest genetic distance was observed between G1 and G2 (0.145) (Table 8).

## 4. Discussion

In this study, 4600 informative DArT markers were used to determine the genetic diversity and population structure of a set of cowpea accessions collected from Togo. Not much has been reported about the genetic diversity of Togolese cowpea germplasm. In the literature, a study on genetic diversity among cowpea germplasm from the country was carried out on 70 accessions using SSR markers [9]. Following the study, the author suggested that cowpea genetic erosion of about 27% was observed in Togo. This, therefore, calls for concerted efforts to collect and preserve cowpea genetic diversity present in the country, especially since Togo is the immediate neighbor to the east of Ghana, both in West Africa. Ghana is where archeological excavations followed by carbon dating of remnant cowpea grains, representing the oldest specimen so far of cultivated cowpea, have been found [44].

Cowpea production across West and Central Africa is mainly in the savanna agro-ecologies due to the crop’s better adaptation to drought prone areas. A similar trend is expected in Togo where there also exist the Region des Savanes and Region Kara, both in dry parts of the country. It is therefore not surprising that germplasm lines collected from the northern part of the country are in the majority while the number from the maritime region (RM) is the lowest. According to production data, RM contributes only 5% of the total cowpea production of Togo [45]. Hence, the lines listed as having been collected from the maritime region were grains most likely obtained from markets in the coastal cities.

In the cowpea germplasm lines used for this study, SNP variation was found to be high with A/G, C/T, A/T, G/T, A/C and G/C having 29.04, 28.13, 11.89, 10.89, 10.11 and 9.94 percent, respectively. The SNPs, A/G and C/T were the most common in this set of cowpea lines. This result confirms earlier studies that showed A/G followed by C/T were most preponderant when compared with the others in cowpea [20,46]. In their study on 768 cowpea cultivars, Xiong et al. [20] found 25.7% and 23.5% A/G and C/T SNPs, respectively, values that are not too different from what we observed. Additionally, the transition/transversion ratio of 1.34 is in agreement with the 1.33 obtained by Ketema et al. [32] in cowpea lines from Ethiopia. According to these authors, the observed ratio might reflect high frequencies of A to G and C to T mutations following methylation. Indeed, sites of cytosine methylation are hot spots for C to T mutations [47].

The expected heterozygosity (0.31) and observed heterozygosity (0.05) for the 255 accessions were comparable to the expected heterozygosity (0.296) and observed heterozygosity (0.075) values reported by Fatokun et al. [21] on 298 cowpea mini-core lines. Similar findings were reported by Xiong et al. [20] for expected heterozygosity, 0.21 to 0.35, and observed heterozygosity of 0.05 to 0.06. Our results also showed that most of the gene diversity was within accessions for RM and ITRA. These results revealed a low level of heterozygotes within the germplasm, which could be explained by the fact that cowpea is a self-pollinated crop with a low level of outcrossing. Although the flower of cowpea is cleistogamous, the occurrence of natural outcrossing could be explained by the activities of visiting insects such as honey and bumble bees, which have been reported to enable outcrossing in cowpea [48]. The decrease of heterozygotes was confirmed by the relatively high mean of F_IS_ (0.83), indicating that most of the accessions are inbred. The range in PIC by region 0.19 (RS) to 0.27 (RM, RP and ITRA) obtained in this study was consistent with gene diversity, which indicated that the accessions from RS were the least variable and those in RM, RP and ITRA most variable. The mean PIC value of 0.25 obtained in this study showed that the markers are reasonably informative. However, when considered by region, the markers were most informative for RM, RP and ITRA (PIC ≥ 0.25) and slightly informative for RS, RK and RC. The mean PIC value in this study was similar to the one obtained by Seo et al. [49], who reported a mean PIC value of 0.287 when assessing Korean cowpea germplasm based on SNP markers.

The analysis of molecular variance (AMOVA) showed that the highest variation (78%) was observed among accessions followed by within accessions (15%) and smallest between populations (7%). These results suggest high genetic variation among accessions within each geographic origin and the accessions from one region to another were almost the same (only 10% of variation). The results confirmed the low to moderate differentiation observed between the populations according to the F_ST_ values (0.014 to 0.117) and confirmed the cluster analysis where accessions did not cluster according to areas of collection. Similar findings have been reported by some other authors. Analyzing the genetic diversity among Senegalese accessions, Sarr et al. [50] reported the largest proportion of variation among accessions (75%), followed by within accessions (14%) and between populations (11%). Additionally, Xiong et al. [51] and Mafakheri et al. [52] reported the highest variation among accessions (90.4% and 77%, respectively) and lowest variation between populations (9.6% and 23%, respectively). According to Chen et al. [53], exploring the genetic diversity of Chinese cowpea germplasm, the results of AMOVA indicated that the majority of the variation occurred within populations, which accounted for 51.6% of the total variation, whereas 25.1% and 23.3% of the variation were attributed to differences within individuals and among populations, respectively. The gene flow among germplasm in our study was higher than 1.0, indicating exchange among cowpea accessions. However, the AMOVA performed on the four clusters showed that the highest variation (45%) was observed among accessions within the clusters followed by between clusters (43%) and smallest within accessions (12%). These results suggest high genetic differentiation among accessions within the clusters and between clusters. The high genetic differentiation is confirmed by the high value for the mean F_ST_ of 0.423 observed between clusters. The gene flow between clusters was less than 1.0, suggesting a limited gene exchange between the four clusters.

The mean F_ST_ value in this study was 0.072 at the regional level showing a moderate differentiation among the germplasm lines. The F_ST_ values between ITRA and each of the two regions (RS and RK) showed moderate differentiation. The moderate differentiation observed between RM and RS, and between RM and RK, could be due to the distance between the regions that might not facilitate easy exchange of seeds. In fact, RM is in the coastal region while RS and RK represent the northern part of the country. The lowest F_ST_ value (0.014) observed between RK and RC suggests that the accessions from the two regions are genetically similar. This relatively low differentiation could be explained by the fact that the two regions are contiguous and seed exchange between their farmers might be high. The majority of pairwise F_ST_ values were lower than 0.05. The genetic differentiation observed by Sarr et al. [50] using 15 SSR markers to assess the genetic diversity among cowpea accessions collected from different regions of Senegal varied from 0.018 to 0.100. A moderate level of genetic differentiation (mean F_ST_ = 0.075) was reported among cowpea accessions collected from Ethiopia also using SSR markers [54]. Kimaro et al. [55] investigated 48 pigeon pea germplasm collections using SSR markers and reported relatively low population differentiation with a mean F_ST_ of 0.032. Like cowpea, pigeon pea is a self-pollinated crop. Its self-pollinating nature has been reported as the reason for the observed low genetic variation among cowpea landraces [56,57]. Yao et al. [58] explained the low differentiation among populations of cowpea as the result of long-distance gene dispersal either by pollen or by seed. The low differentiation generally observed among cowpea reflects the initial bottleneck during domestication, which is maintained by the inherent self-pollination mechanism in the crop [59].

Cluster analysis classified cowpea accessions into four major clusters, which can also be detected on the PCoA plot along the first two principal coordinates. In the present study, the accessions clustered together irrespective of the region from where they were collected. This could be attributed to farmer-to-farmer seed exchange, a very common practice in SSA.

The structure analysis showed that the 255 accessions belong to two gene pools that could be further divided into four gene pools. This observation is in agreement with the findings from an earlier study of diversity among 422 cowpea landraces collected from about 56 countries, following genotyping with a 1536-SNP GoldenGate assay [46]. It is worth noting that a diversity study carried out on 95 accessions of yard-long-bean from China belong to two gene pools [60]. The long-podded yard-long-bean (*V. unguiculata* var. *sesquipedalis*) is the type of cowpea commonly grown and consumed as a vegetable in Asia. It has been suggested that this vegetable type cowpea originally came from Africa, after which it acquired its present characteristics following being grown in more humid environments with lower light intensity, unlike in SSA, as well as selection by farmers for succulent pods [46].

## 5. Conclusions

This study revealed the existence of genetic diversity among cowpea germplasm collections from Togo. The four groups delineated by cluster analysis were in agreement with the four gene pools identified by population structure. The grouping did not bear close relationships with the geographical regions of collection. This detection of four groups among accessions from Togo agree with earlier findings on cowpea germplasm accessions from other countries. The results of this molecular characterization would be relevant to cowpea improvement efforts in Togo and serve as reference for selecting accessions with desirable traits for breeding purposes. The genetic diversity detected within the germplasm would be of benefit to a cowpea improvement program in the country.

## Figures and Tables

**Table 1:** Percentage of DArT SNP type used in this study.

SNP Type	Number	Proportion	Percentage
A/G	1336	0.2904	29.04
C/T	1294	0.2813	28.13
A/T	547	0.1189	11.89
G/T	501	0.1089	10.89
A/C	465	0.1011	10.11
C/G	457	0.0994	9.94
Total	4600	1.0000	100.00

**Table 2:** Mean value of MnAF, MAF, He, Ho, PIC and F_IS_ among the populations.

	MnAF	MAF	He	Ho	PIC	F_IS_
RS	0.15	0.85	0.22	0.05	0.19	0.79
RK	0.18	0.82	0.26	0.03	0.21	0.89
RP	0.24	0.76	0.33	0.06	0.27	0.82
RM	0.26	0.74	0.34	0.06	0.27	0.84
RC	0.21	0.79	0.29	0.05	0.24	0.84
ITRA	0.25	0.75	0.34	0.07	0.27	0.78
Germplasm	0.22	0.78	0.31	0.05	0.25	0.83

MnAF = minor allele frequency, MAF = major allele frequency, He = gene diversity or expected heterozygosity, Ho = observed heterozygosity, PIC: polymorphism information content, F_IS_ = inbreeding coefficient, RS = Region des Savanes, RK = Region de la Kara, RP = Region des Plateaux, RM: Region Maritime, RC = Region Centrale, ITRA = Institut Togolais de Recherche Agronomique.

**Table 3:** Analysis of molecular variance among 255 accessions assessed with 4600 SNP markers.

Source	df	SS	MS	Est. Var.	PV	F-Statistics	*p*-Value
Among Pops	5	29,485.158	5897.032	56.185	7%	F_ST_ = 0.072	0.001
Among Indiv	249	329,937.822	1325.051	603.339	78%	F_IS_ = 0.836	0.001
Within Indiv	255	30,185.000	118.373	118.373	15%	F_IT_ = 0.848	0.001
Total	509	389,607.980		777.897	100%		
Nm	3.21						

df: degree of freedom, SS: sum of squares, MS: mean square, Est. Var.: estimated variance, PV = percentage variance, F_ST_: genetic differentiation, F_IS_: fixation index or inbreeding coefficient, F_IT_: overall fixation index, Nm: gene flow.

**Table 4:** Pairwise population F_ST_ values.

	RS	RK	RP	RM	RC	ITRA
RS	0.000					
RK	0.017	0.000				
RP	0.055	0.032	0.000			
RM	0.117	0.091	0.032	0.000		
RC	0.032	0.014	0.023	0.067	0.000	
ITRA	0.067	0.055	0.030	0.035	0.036	0.000

RS: Region des Savanes, RK: Region de la Kara, RP: Region des Plateaux, RM: Region Maritime, RC: Region Centrale, ITRA: Institut Togolais de Recherche Agronomique.

**Table 5:** Analysis of molecular variance among 255 accessions assessed with 4600 SNP markers.

Source	df	SS	MS	Est. Var	PV	F-Statistics	*p*-Value
Among Pops	3	126,471.501	42157.167	383.888	42%	F_ST_ = 0.423	0.001
Among Indiv	251	232,951.480	928.094	404.860	45%	F_IS_ = 0.774	0.001
Within Indiv	255	30,185.000	118.373	118.373	13%	F_IT_ = 0.870	0.001
Total	509	389,607.980		907.121	100%		
Nm	0.381						

**Table 6:** Pairwise F_ST_ values among clusters.

	Cluster I	Cluster II	Cluster III	Cluster IV
Cluster I	0.000			
Cluster II	0.160	0.000		
Cluster III	0.209	0.290	0.000	
Cluster IV	0.177	0.173	0.140	0.000

**Table 7:** Number of accessions assigned to the four gene pools and admixture.

**Population**	**G1**	**G2**	**G3**	**G4**	**Admixture**
RS	2	47	0	6	17
RK	6	30	4	1	7
RP	6	23	12	1	18
RM	0	3	7	0	8
RC	4	10	3	1	3
ITRA	3	8	6	5	14
Total	21	121	32	14	67

**Table 8:** Genetic divergence (net nucleotide distance) among the gene pools.

	**G1**	**G2**	**G3**	**G4**
G1	0.000			
G2	0.145	0.000		
G3	0.254	0.320	0.000	
G4	0.289	0.260	0.265	0.000

## Data Availability

The DArT genotypic data generated for this study has been provided as a supplementary material (File S1).

## Supplementary Materials

The following are available online at https://www.mdpi.com/article/10.3390/genes12091451/s1, File S1: DArTseq data, Figure S1: Map of Togo showing locations of the accessions.
