# Supplementary figures and images for: Genetic Diversity and Population Structure of Cowpea [Vigna unguiculata (L.) Walp.] Germplasm Collected from Togo Based on DArT Markers
Source: Genes (Basel). 2021 Sep 20;12(9):1451. doi: 10.3390/genes12091451 (PMC8465771; doi:10.3390/genes12091451)

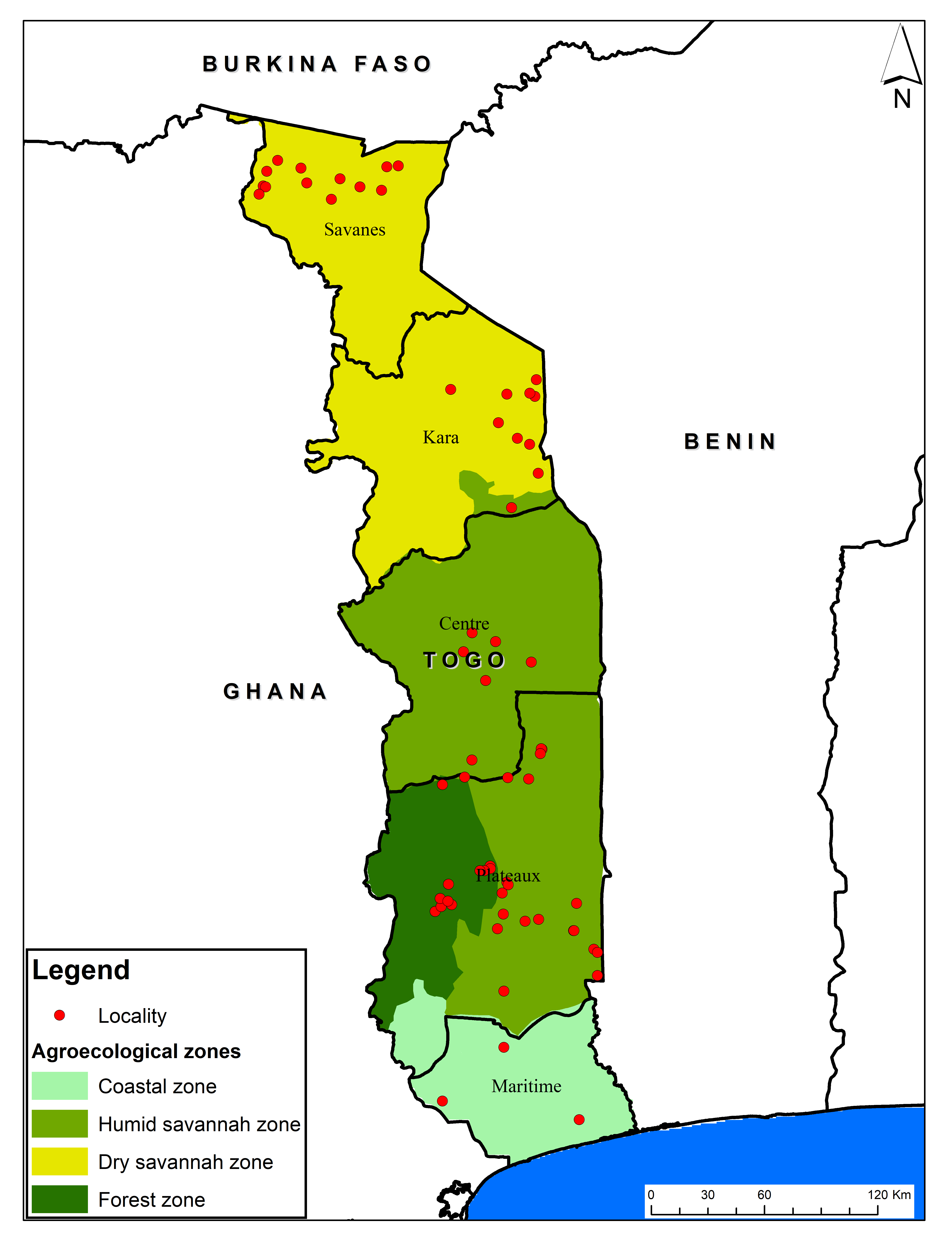

Supplement: Supplementary file 1 [file genes-12-01451-s001.zip › Figure S1.tif]
